# Evidence of genetic isolation between two Mediterranean morphotypes of *Parazoanthus axinellae*

**DOI:** 10.1038/s41598-020-70770-z

**Published:** 2020-08-18

**Authors:** Adriana Villamor, Lorenzo F. Signorini, Federica Costantini, Marko Terzin, Marco Abbiati

**Affiliations:** 1 Dipartimento di Scienze Biologiche, Geologiche ed Ambientali (BiGeA) & Centro Interdipartimentale di Ricerca per le Scienze Ambientali (CIRSA), Università di Bologna, Via S. Alberto 163, 48123 Ravenna, Italy; 2 International Council for the Exploration of the Sea, HC Andersen 22-24, 1553 Copenhagen, Denmark; 3 Faculty of Life Sciences, Tel Aviv University, Ramat Aviv, 69978 Israel; 4 CoNISMa, Piazzale Flaminio 9, 00196 Rome, Italy; 5 Consiglio Nazionale Delle Ricerche, Istituto di Scienze Marine, ISMAR, Bologna, Italy; 6 Dipartimento di Beni Culturali, Università di Bologna, Via degli Ariani, 1, 48121 Ravenna, RA Italy

**Keywords:** Molecular ecology, Ecology, Biodiversity

## Abstract

Coralligenous assemblages are among the most species-rich and vulnerable habitats of the Mediterranean Sea. Nevertheless, data on connectivity patterns on species inhabiting these habitats, crucial to define management and protection priorities, are largely lacking. Moreover, unreliable species-level taxonomy can confound ecological studies and mislead management strategies. In the northwestern Mediterranean two *Parazoanthus axinellae* morphotypes differing in size, color and preferred substrate are found in sympatry. In this study, we used COI and ITS sequence polymorphism to assess (1) the genetic divergence between the two morphotypes, (2) their connectivity patterns and (3) their phylogenetic position within the Parazoanthidae. Specimens of *P. axinellae* were sampled in 11 locations along the northwestern Mediterranean; in 6 locations, samples of the two morphotypes were collected in sympatry. Small genetic diversity and structure were found within morphotypes, while marked and consistent differentiation was detected between them. Moreover, the less widespread morphotype appeared to be closer to Pacific species as *P. juanfernandezii* and *P. elongatus*. Our findings confirmed the limited knowledge on *Parazoanthus* species complex, and how this gap can have important implication for the conservation strategies of this widespread and valuable genus in the Mediterranean Sea.

## Introduction

Biogenic reefs are made by calcareous encrusting algae and animals that change the geological primary habitat on which they settled through superimposition of their skeletons. They are among the most productive and diverse benthic ecosystems, providing habitat, feeding grounds, recruitment and nursery areas for a variety of invertebrate and vertebrate species^1^.

In the Mediterranean Sea, the main biogenic reefs are the coralligenous reefs. They are among the most important Mediterranean ecosystems due to the high diversity of species and ecological processes that they support^1,2^. These complex habitats are threatened by several human activities (e.g. recreational fishing and trawling, sediment deposition, anchorage, diving), which lead to their fragmentation and loss^3,4^.

Monitoring spatio-temporal changes in species composition is crucial to quantify human-induced biodiversity loss and habitat fragmentation, but the absence of clear taxonomic identifiers to distinguish between species makes this a challenging task.

Many relevant coralligenous taxa (e.g. Porifera, Echinodermata, Cnidaria) lack diagnostic morphological characteristics, and therefore these groups are recognized as taxonomically problematic, in particular at lower taxonomic levels^5^. Moreover, in these species, with high morphological plasticity^6^, some characters such as shape and coloration might not be reliable for species delimitation. It happened that morphospecies have turned out to be a single morphologically variable species^7–9^, and, conversely, what was thought to be multiple growth forms of a single species turned out to be a complex of species^10,11^.

The presence of species complexes in the sea can confound ecological studies and mislead management strategies. For example, connectivity patterns^12^ may be erroneously inferred when cryptic species are ignored^13,14^, which may provide biased conclusions about the overall capacity of the studied populations to resist stress^15^. To disclose cryptic species before estimating gene flow and indeed evaluate connectivity among populations, we should (1) use more than one independent molecular marker, (2) sample individuals all along the distributional range of the investigated species, and (3) sample closely related taxonomic species to understand the spectrum of population to species divergence^13^.

**Figure 1:**
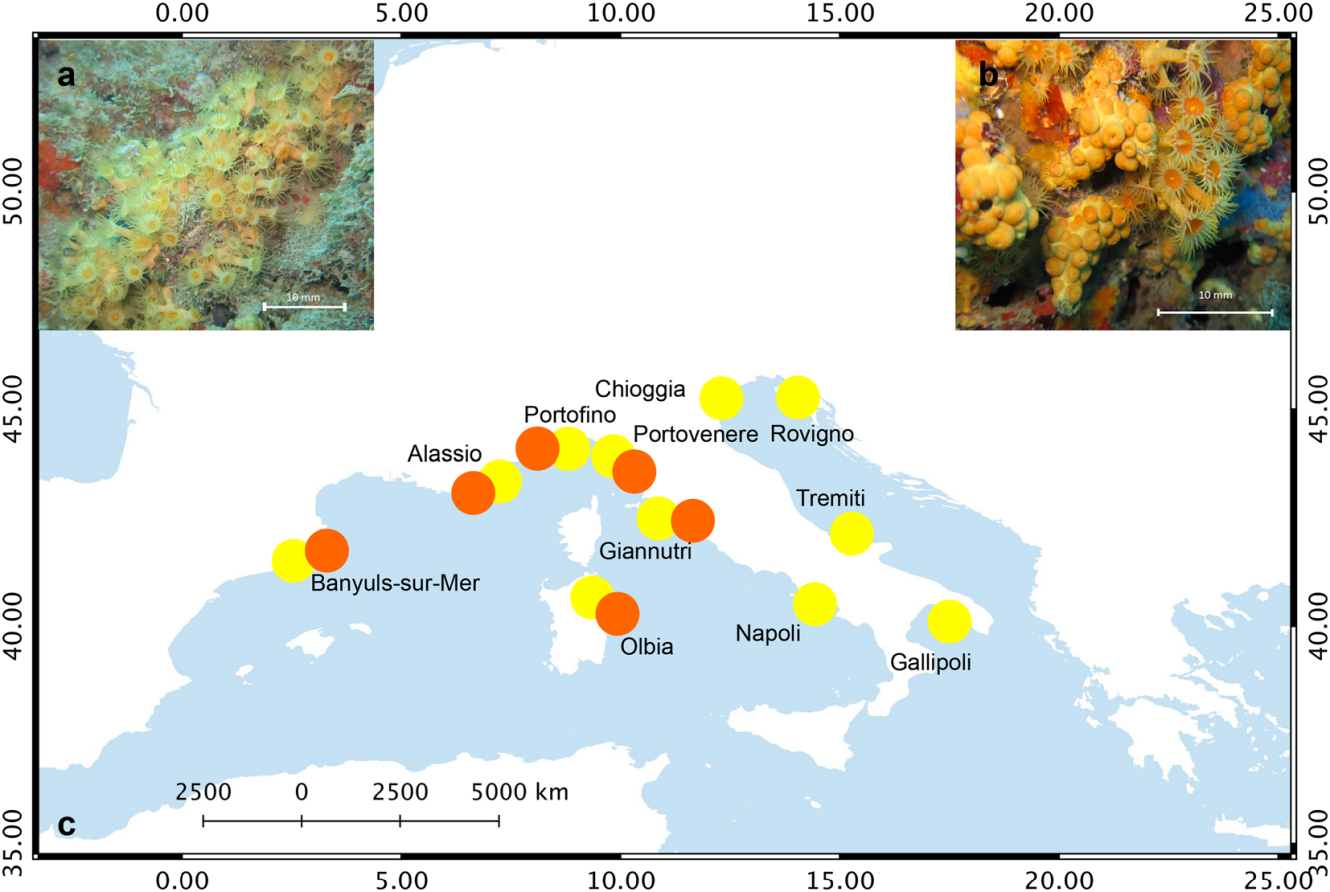
(**a**) “Slender” morphotype of *Parazoanthus axinellae* (Photo: A. Villamor); (**b**) “Stocky” morphotype of *Parazoanthus axinellae*, secca del Tinetto, Portovenere, Italy (Photo: A. Villamor); (**c**) map of the sampling sites where the colonies of *P. axinellae* were collected. Yellow dots represent samples of “Slender” morphotype; orange dots represent samples of “Stocky” morphotype*,* The map was created with the free software QGIS (https://qgis.osgeo.org/es/site/) and edited in Adobe Photoshop version 14.2.1 (www.adobe.com) for this study.

To our knowledge, three species of *Parazoanthus* exist along the Mediterranean and North-East Atlantic coasts: *Parazoanthus axinellae* (Schmidt, 1862), *P. anguicomus* (Norman, 1869) and another *Parazoanthidae* yet to be described in the Macaronesian waters^16^. In the Mediterranean Sea, *Parazoanthus axinellae* (Cnidaria, Hexacorallia, Zoantharia) species complex^17^ is one of the most common cnidarians inhabiting coralligenous assemblages. The simplicity of the zoanthid body plan makes morphology-based species identification quite challenging. Numerous morphological identification criteria have been used to identify phylogenetic signal ranging from colour, sphincter muscle anatomy^18^, tentacles number^19^, type and distribution of nematocysts^20^. In addition, differences in substrate preference, overall ecology^17^, biochemical profiles^21–23^ and sequence divergence using various gene markers were also proposed as zoanthid identification criteria^24–26^. Original species descriptions were thereby based on different traits depending on the authors^27^. Due to the aforementioned lack of morphological characters to identify zoanthids, a comprehensive taxonomic identification of the *P. axinellae* species complex is still missing (but see^17^ for a detailed description of *P. axinellae* species complex). Historically, there were four described subspecies of *P. axinellae: P. axinellae mülleri* Pax, 1957, common across the whole Mediterranean coastline*; P. axinellae liguricus* Pax, 1937, in the Ligurian Sea*; P. axinellae brevitentacularis* in the Gulf of Lion and *P. axinellae adriaticus* Pax, 1937 in the Adriatic Sea^28–30^. However, the morphological descriptions of these four subspecies are quite imprecise and overlapping, and clear diagnostic characters to discriminate them are still lacking. Recently, Ocana et al.^17^ stated that these four subspecies can be grouped in two morphotypes based on their chemical profiles^31^ and ecological requirement^17^. These two morphotypes live in sympatry in the northwestern Mediterranean^29^. One of them displays an elongated trunk, longer and thinner tentacles, and a light yellow color. This morphotype (“Morphotype 1” in^17^, “Slender” in^31^; Fig. 1a) is found across the whole Mediterranean, including the Adriatic Sea, and could correspond to the descriptions of the two subspecies *P. axinellae mülleri* and *P. axinellae adriaticus* (Fig. 1a). A more pronounced orange color, and shorter and thicker trunk and tentacles characterize the second morphotype (Morphotype 2 in^17^, “Stocky” in^31^; Fig. 1b). This form is restricted to the north-western Mediterranean and could correspond to *P. axinellae brevitentacularis* and *P. axinellae liguricus* (Fig. 1b). On top of their morphological differences, the “Slender” form is mainly characterized by an epibiotic lifestyle on the sponges of the genus *Axinella*, while the “Stocky” form lives primarily on the rocky substratum^32^ and has never been observed on *Axinella* spp^17^.

In the present study, we aim to describe the patterns of diversity and connectivity for populations of the two *Parazoanthus axinellae* morphotypes along the Northwestern Mediterranean and the Adriatic Sea. Specifically, we use mitochondrial (COI) and nuclear (ITS) sequences polymorphism to asses: (1) the genetic diversity and structure among populations within and between morphotypes and (2) phylogeny and differentiation in the Parazoanthidae Family.

## Results

### Genetic diversity and structure among *P. axinellae* populations

**Figure 2:**
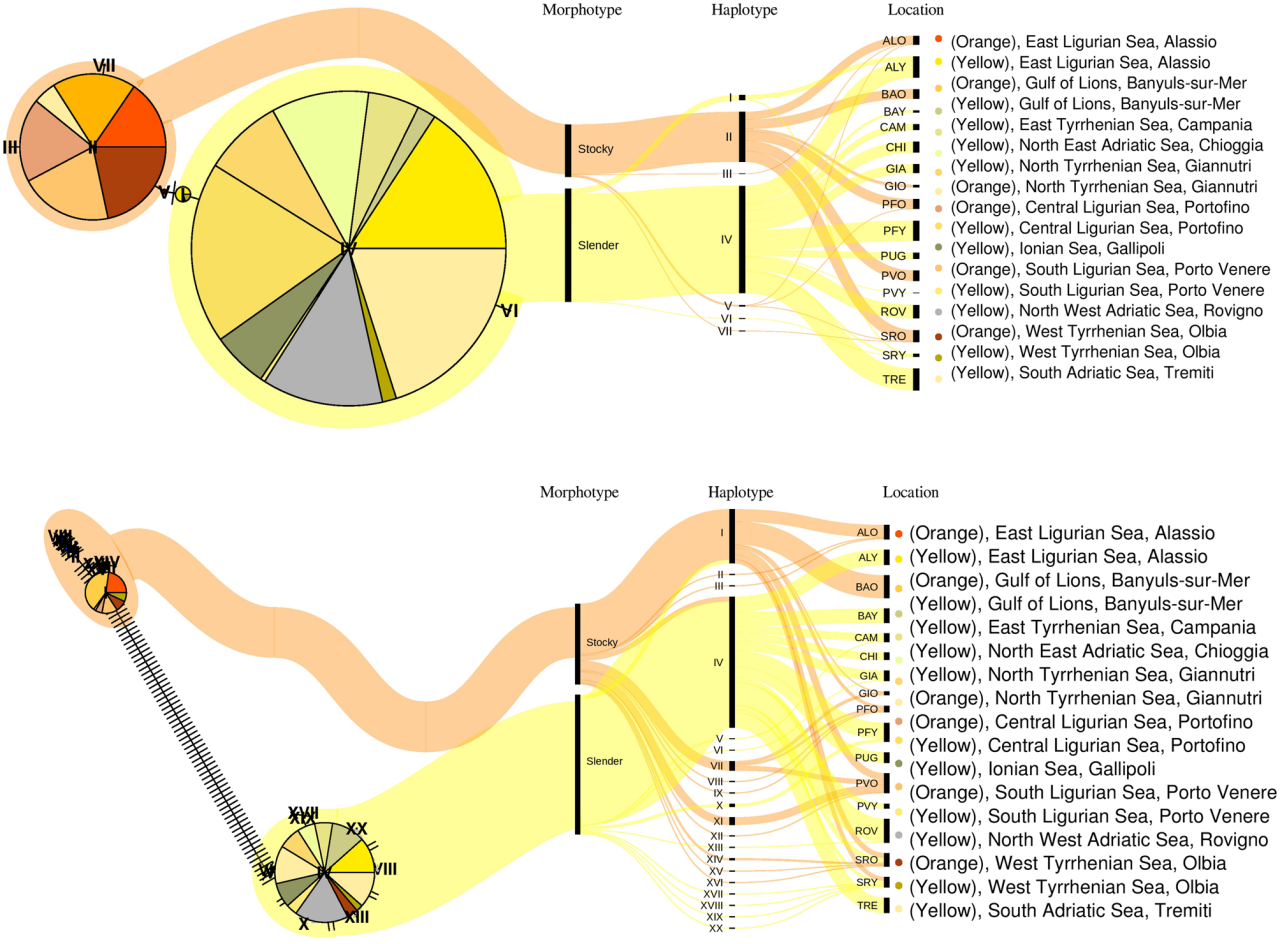
Haplotype network analysis for COI (above) and ITS (below) gene markers in *Parazoanthus axinellae* species complex. Haplotype network was built in Rstudio version 1.1.453 (https://rstudio.com/) with an R package *pegas* (https://cran.r-project.org/web/packages/pegas/index.html), using an infinite site model based on Hamming distances of DNA sequences. Each haplotype is presented as a separate pie chart, and size of the pie charts directly correlates to the number of individuals within the haplotype. Pie chart colors for geographic location were chosen to intuitively associate the reader to *Parazoanthus axinellae* morphotypes, with orange tones being attributed to the “Stocky” morphotype, and yellow and grey tones to the “Slender” morphotype. Distances between haplotypes correspond to the genetic differentiation observed for each of the markers, and each mutation between the haplotypes is shown as a hyphen. Alluvial diagrams were made using an online platform RAWGraphs (https://rawgraphs.io/) to further clarify haplotype-morphotype assignments, and final graph modifications were done in Inkscape version 0.92.4 (https://inkscape.org/it/).

A total of 322 sequences of 402 bp of COI were obtained (Table 1), and 5 polymorphic sites and 7 haplotypes were detected. Haplotype and nucleotide diversity were low within all the localities. The “Slender” and the “Stocky” morphotypes showed 3 (I, IV, VI) and 4 (II, III, V, VII) different haplotypes, respectively (Fig. 2, Supplementary Figure S1, Supplementary Table S1). The unrooted haplotype network exhibited two star-like patterns (haplogroups: “Slender” and “Stocky”) connected by 1 mutational step. The two haplogroups were surrounded by private and low frequency haplotypes differing in 1 bp (Fig. 2, Supplementary Figure S1, Supplementary Table S1).

**Table 1:** Locality, geographical coordinates, morphotype, depth, and code of *Parazoanthus axinellae* samples; number of individuals sequenced (n), haplotypes (H), haplotype (Hd) and nucleotide (π) diversity values per location (with their standard deviation—SD) for both molecular markers.

Locality	Coordinates	Morphotype	Depth	Code	n_COI_	H_COI_	Hd_COI_	SD	π_COI_	SD	n_ITS_	H_ITS_	Hd_ITS_	SD	π_ITS_	SD
Gulf of Lions, Banyuls-sur-Mer	42.6955, 3.3216	Slender	15	BAY	4	1	0	0	0	0	15	1	0	0	0	0
Gulf of Lions, Banyuls-sur-Mer	42.6955, 3.3216	Stocky	15	BAO	18	1	0	0	0	0	24	1	0	0	0	0
East Ligurian Sea, Alassio	44.0226, 8.2641	Slender	17	ALY	41	2	0.322	0.076	0.00081	0.00019	16	1	0	0	0	0
East Ligurian Sea, Alassio	44.0226, 8.2641	Stocky	17	ALO	17	3	0.228	0.129	0.00059	0.00035	15	3	0.257	0.142	0.00279	0.00149
Central Ligurian Sea, Portofino	44.2983, 9.2184	Slender	15	PFY	39	1	0	0	0	0	20	2	0.268	0.113	0.00046	0.00019
Central Ligurian Sea, Portofino	44.2983, 9.2184	Stocky	15	PFO	19	2	0.105	0.092	0.00026	0.00023	7	4	0.81	0.130	0.0087	0.00153
South Ligurian Sea, Porto Venere	44.0227, 9.8511	Slender	15	PVY	1	1	0	0	0	0	5	1	0	0	0	0
South Ligurian Sea, Porto Venere	44.0227, 9.8511	Stocky	15	PVO	20	1	0	0	0	0	21	4	0.719	0.047	0.0068	0.00073
North Tyrrhenian Sea, Giannutri	42.2387, 11.1035	Slender	25	GIA	17	1	0	0	0	0	11	2	0.182	0.144	0.00062	0.00049
North Tyrrhenian Sea, Giannutri	42.2387, 11.1035	Stocky	25	GIO	5	1	0	0	0	0	4	2	0.5	0.265	0.0069	0.00366
West Tyrrhenian Sea, Olbia	40.9999, 9.6606	Slender	16	SRY	6	3	0.733	0.155	0.00217	0.00062	11	6	0.836	0.089	0.06679	0.01254
West Tyrrhenian Sea, Olbia	40.9999, 9.6606	Stocky	16	SRO	23	3	0.170	0.102	0.00044	0.00027	14	5	0.769	0.076	0.06451	0.01093
East Tyrrhenian Sea, Campania	40.5873, 14.3760	Slender	15	CAM	11	1	0	0	0	0	9	2	0.222	0.166	0.00038	0.00028
Ionian Sea, Gallipoli	40.1444, 17.9719	Slender	35	PUG	12	1	0	0	0	0	11	1	0	0	0	0
South Adriatic Sea, Tremiti Is	42.1354, 15.5085	Slender	20	TRE	42	1	0	0	0	0	16	1	0	0	0	0
North East Adriatic Sea, Chioggia	45.2110, 12.3839	Slender	18	CHI	21	1	0	0	0	0	8	1	0	0	0	0
North West Adriatic Sea, Rovigno	45.0513, 13.6232	Slender	13	ROV	26	1	0	0	0	0	25	2	0.08	0.072	0.00014	0.00012
ALL SLENDER		Slender			220	3	0.096	0.027	0.00024	0.00007	147	10	0.181	0.043	0.00714	0.00324
ALL STOCKY		Stocky			102	4	0.096	0.04	0.00024	0.0001	85	12	0.59	0.058	0.01954	0.00549

As for the ITS fragment, a total of 20 haplotypes were obtained from 232 sequences (698 bp) and “Stocky” and “Slender” morphotypes were differentiated by a minimum number of 68 mutation steps (between haplotypes II and VI) (Fig. 2, Supplementary Figure S1, Supplementary Table S2). The two morphotypes showed different ITS haplotypes, with the exception of 9 individuals sampled in Olbia, West Tyrrhenian Sea (codes SRY and SRO) that shared the same haplotype with the morphologically distinct phenotype. In particular, 5 “Stocky” individuals at Olbia (code SRO) had the identical ITS sequence with the haplotype IV (mostly composed out of “Slender” individuals), and 4 specimens with the “Slender” phenotype (code SRY) shared the same sequence with haplotype I, composed mostly of “Stocky” *Parazoanthus* specimens (see Fig. 2, Supplementary Figure S1).

Genetic differentiation (pairwise Φ_ST_ values) between populations ranged between 0 and 1 for both markers (See Supplementary Table S3, Supplementary Table S4; Supplementary Figure S2, Supplementary Figure S3). The lowest differentiation values corresponded to the comparisons between populations of the same morphotype, whereas the highest differentiation values occurred between populations of different morphotypes. Significant differentiation was also detected (for ITS) between populations within the “Stocky” morphotype, in particular between population pairs Banyuls-sur-Mer/Alassio and Porto Venere/Portofino/Giannutri (Φ_ST BAO versus PVO_ = 0.60; Φ_ST BAO versus PFO_ = 0.70; Φ_ST ALO versus PVO_ = 0.37; Φ_ST ALO versus PFO_ = 0.35; Φ_ST BAO versus GIO_ = 0.99; Φ_ST ALO versus GIO_ = 0.58) (Supplementary Table S4 Supplementary Figure S3). The samples from Olbia (SRY and SRO codes) showed a specific pattern of genetic differentiation, with ITS haplotypes being intermixed within morphotypes, as previously elaborated.

The Analysis of Molecular Variance (AMOVA) between groups of samples according to morphotype and biogeographic areas showed that for the “Slender” morphotype most variability is explained by differences between all populations (COI: 81.79%, *P* < 0.01; ITS: 68.87%, *P* < 0.01; Table 2). For the “Stocky” morphotype, the same is true according to COI, but ITS shows some variability related to the biogeographic pattern (Table 2).

**Table 2:** Results of the analysis of molecular variance (AMOVA). For each marker groups were defined according to biogeographic areas (A) within the “Slender” morphotype; (B) within the “Stocky” morphotype and (C) between morphotypes (d.f.: degrees of freedom; % var: percentage of variation)

Source of variation	COI				ITS			
	d.f	Variance components	%	*P* value	d.f	Variance components	%	*P* value
**(A)**								
Within “Slender”	4 biogeographic areas				4 biogeographic areas			
Among locations	3	− 0.004	− 8.86		3	− 0.087	− 2.45	0.372
Among samples within locations	7	0.013	27.08		7	1.191	33.58	0.010
Within samples	204	0.040	81.79		136	2.442	68.87	0.000
**(B)**								
Within “Stocky”	2 biogeographic areas				3 biogeographic areas			
Among locations	1	0	− 0.63		2	2.716	24.97	0.061
Among samples within locations	4	0	− 1.18		3	52.11	12.61	0.053
Within samples	96	0.048	101.81		79	4.07	62.42	0.000
**(C)**								
Between morphotypes								
Among morphotypes	1	1.420	96.62		1	51.37	89.52	0.000
Among samples within morphotypes	15	0.005	0.36		15	1.93	3.37	0.000
Within samples	300	13.348	3.03		215	4.07	7.10	0.000

AMOVA between the two groups defined by the morphotype attributed most of the variation to the differences between morphotypes for both markers (COI: 96.62%, *P* < 0.01; ITS: 89.52%, *P* < 0.01; Table 2).

Neutrality tests showed no departure from neutrality according to COI in both morphotypes, but a positive and significant departure according to *Fu and Li* test^33^ for the ITS marker in both morphotypes (1.77, *P* < 0.02 for the “Slender”; and 2.28 *P* < 0.02 for the “Stocky”), which might indicate a decrease in population size and/or balancing selection acting in this marker. Mismatch distributions in both markers showed a bimodal distribution^34^, but the observed distributions were not statistically different from those expected under a sudden expansion model (COI: SSD = 0.15 *P* value = 0.047; rg = 0.48 *P* value = 0.05; ITS: SSD = 0.09 *P* value = 0.22; rg = 0.30 *P* value = 0.42). Two genetic pools were observed; one is corresponding to a recent expansion and a second one with older demographic history. These two genetic pools are more similar according to COI, but very distant according to ITS (Supplementary Figure S4, Supplementary Figure S5).

### Phylogeny and differentiation within Family Parazoanthidae

Haplotype function from *pegas*^35^ dereplicated identical sequences into a total number of 16 (COI) and 38 (ITS) haplotypes within Parazoanthidae family (sequences from our study + those retrieved from GenBank) (Supplementary Table S5, Supplementary Table S6). These haplotypes were reconstructed from 344 (COI) and 275 (ITS) same-length sequences (474 and 734 nucleotides for COI and ITS genes) and were used for Maximum Likelihood (ML) treebuilding.

Likelihood ratio test (performed with modelTest function in *phangorn* R package), based on minimum AICc values, identified HKY + I and HKY + G + I models as best nucleotide evolution models for COI and ITS markers, respectively. The Maximum Likelihood trees were reconstructed for each marker using F81 nucleotide substitution model as this was the most similar model to HKY + I and HKY + G + I available in *phangorn* R package.

Although indels in ITS gene marker created large gaps, the overall network topology and the number of mutation steps between species did not vary greatly between the different MAFFT alignments performed (only one result is shown). Moreover, the phylogenetic trees with MAFFT alignment (without gap removal) and with MAFFT alignment + GBlock gap removal also give similar topology. We only show results for *P. axinellae* + Genbank ITS sequences aligned in MAFFT using L-INS-i algorithm with 1,000 iterations, and with Gblock gap removal parameters set to: b1 = 0.8, b2 = 0.9, b4 = 2, b5 = "h".

**Figure 3:**
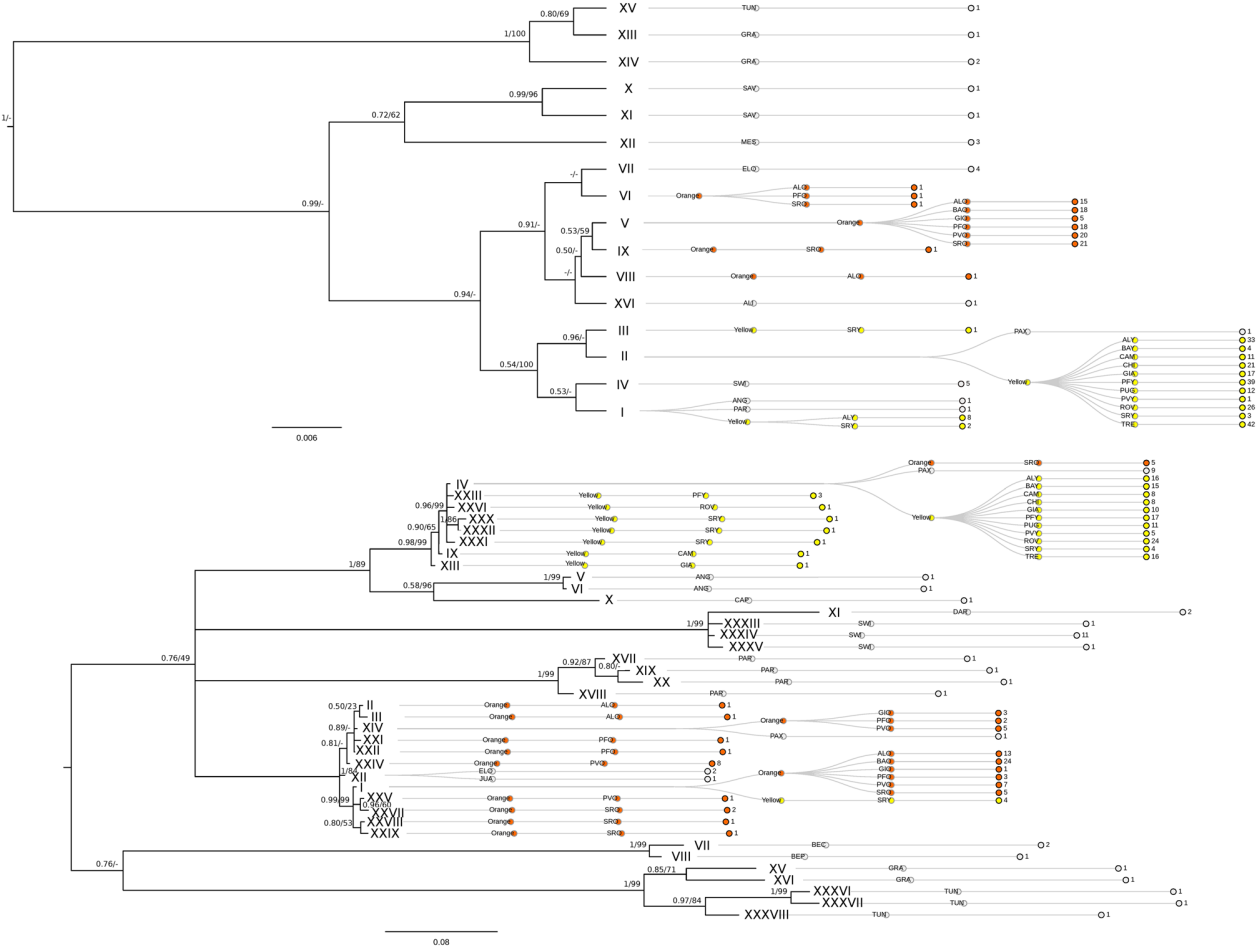
Phylogenetic trees and clustering dendrograms based on COI (above) and ITS (below) gene markers between 16 (COI) and 38 (ITS) haplotypes within Parazoanthidae family, respectively. Identical sequences were dereplicated into haplotypes and are presented on the tree nodes in roman numerals. For ITS the phylogentic tree using the MAFFT + GBlock alignment is shown. Bayesian posterior probability (on the left) and Maximum Likelihood (ML) bootstrap support based on 1,000 iterations (on the right) are shown on tree branches with a cutoff value of *p* = 50 (%). Inferior support values were considered as unresolved. Clustering dendrograms were reconstructed for each gene marker in RAWGraphs (https://rawgraphs.io/) and added as extensions onto tree nodes to further clarify haplotype/sequence assignments. Clustering dendrograms hierarchically consist out of 2 levels for sequences downloaded from GenBank (Species code, Number of sequences) and 3 levels for samples sampled in our study (Morphotype Color, Sampling Location, Number of sequences). Sequences originating from our samples are colored in yellow and orange for “Slender” and “Stocky” morphotypes, respectively, while all sequences downloaded from GenBank are in grey. Codes are as follows for *Parazoanthus* sp. sequences from NCBI: ALI—*Parazoanthus aliceae*, NE Atlantic; ANG—*Parazoanthus anguicomus*, NE Atlantic; BEC—*Bergia catenularis*, Carribean Sea; BEP—*Bergia puertoricense*, Carribean Sea; CAP—*Parazoanthus capensis*, Port Elizabeth, South Africa; DAR—*Parazoanthus darwini*, Galapagos islands; ELO—*Parazoanthus elongatus*, multiple localities; GRA—*Hydrozoanthus gracilis*, multiple localities; JUA—*Parazoanthus juanfernandezii*, South Pacific; MES—*Mesozoanthus fossii,* South Pacific; PAX—*Parazoanthus axinellae*, multiple localities; PAR—*Umimayanthus parasiticus*, Caribbean Sea; SAV—*Savalia savaglia*, Gran Canaria (Spain); SWI—*Parazoanthus swiftii*, multiple localities; TUN—*Hydrozoanthus tunicans*, multiple localities. Codes for individuals of *P. axinellae* species complex sampled in our study are as follows: ALO—(Orange), East Ligurian Sea, Alassio; ALY—(Yellow), East Ligurian Sea, Alassio; BAO—(Orange), Gulf of Lions, Banyuls-sur-Mer; BAY—(Yellow), Gulf of Lions, Banyuls-sur-Mer; CAM—(Yellow), East Tyrrhenian Sea, Campania; CHI—(Yellow), North East Adriatic Sea, Chioggia; GIA—(Yellow), North Tyrrhenian Sea, Giannutri; GIO—(Orange), North Tyrrhenian Sea, Giannutri; PFO—(Orange), Central Ligurian Sea, Portofino; PFY—(Yellow), Central Ligurian Sea, Portofino; PUG—(Yellow), Ionian Sea, Gallipoli; PVO—(Orange), South Ligurian Sea, Porto Venere; PVY—(Yellow), South Ligurian Sea, Porto Venere; ROV—(Yellow), North West Adriatic Sea, Rovigno; SRO—(Orange), West Tyrrhenian Sea, Olbia; SRY—(Yellow), West Tyrrhenian Sea, Olbia; TRE—(Yellow), South Adriatic Sea, Tremiti.

Bayesian Inference and maximum likelihood trees reflected the same topology for both COI and ITS genes but with different statistical support. It is interesting to note that various haplotypes in both markers shared the same sequence across more than one species. Particularly for COI gene, this was the case for haplotypes I (ANG, PAR and “Slender” *P. axinellae*), and V (ALI and “Stocky” *P. axinellae*), whereas for ITS marker this occurred only in haplotype VI (ELO and JUA). Overall “Stocky” and Slender” morphotypes cluster in two well-separated clades (although with low bootstrap support). In the “Stocky” clade all the “Stocky” morphotype haplotypes showed high similarity with *P. elongatus* (COI and ITS)*,* and *P. juanfernandezii* (ITS), both species from the southeastern Pacific. The “Slender” morphotypes grouped with *P. anguicomus* (COI and ITS) from the eastern Atlantic, *P. parasiticus* (COI), *P. swiftii* (COI) both Caribbean species and *P. aliceae* (COI) from the Azores Archipelago (Fig. 3). Finally, it is interesting to note that despite clear genetic differences between the studies morphotypes, certain individuals sampled in Olbia, West Tyrrhenian Sea (codes SRY and SRO) shared the same sequence for ITS marker with the morphologically distinct phenotype (Fig. 3).

## Discussion

In the present study we evidenced for the first time that (1) the two Mediterranean morphotypes of *Parazoanthus axinellae* differ more between them that in comparison to other allopatric zoanthid species and (2) that the “Slender” morphotype is characterized by a lower genetic differentiation among populations compared to the “Stocky” morphotype.

COI and ITS evidenced clear genetic isolation between the two morphotypes. Differences in chemical profiles^31^ and in morphological features^17^ between the two morphotypes are consistent with our results, suggesting that the *Parazoanthus axinellae* species complex includes two different taxa. Both COI and ITS haplotype networks showed the presence of two highly abundant haplotypes surrounded by low frequency haplotypes that clearly discriminate the two morphotypes. This low variability can be explained by the geographical distribution of the morphotypes. In fact, the “Slender” morphotype is widespread all along the Mediterranean Sea while the “Stocky” morphotype^17^ has a more restricted distribution. Although we cannot discard the existence of more populations of the “Stocky” morphotype in other unsampled areas of the Atlantic Ocean, information on the geographic distribution of this species in the Eastern Atlantic Ocean is scarce and refers only to the “Slender” morphotype (Boavida J., personal communication). The widespread distribution of the “Slender” morphotype can also explain the low genetic structuring observed among populations.

Despite the limited geographical distribution compared to the “Slender” morphotype, the “Stocky” morphotype shows genetic structure between northeastern Tyrrhenian populations (Portovenere, Portofino, and Giannutri) and the northwestern ones (Banyuls -sur -mer and Alassio) with a break around Portofino. Portofino area represents a barrier to gene flow for several species of sessile invertebrates (*Corallium rubrum*^36^; *Paramuricea clavata*^37^; *Patella caerulea*^38^) related to the presence of a large‐scale separation of currents that occurs in the region^39^. Biological and reproductive characteristics of the species can also contribute to explain the observed pattern. Pax and Muller^40^ found hermaphroditic colonies of *P. axinellae* in the Adriatic Sea, while observed that colonies of *P. axinellae* from the British Isles (North Atlantic) are gonochoric^41^. Moreover, Previati et al.^42^ observed that the “Stocky” morphotype collected in the Ligurian Sea, where the two morphotypes live in sympatry, exhibited both asexual and sexual reproduction (Cerrano C. personal communication). Although not much information is available on the ecological and biological traits of this morphotype, it seems to be defined by a lower dispersal capacity compared to the “Slender” morphotype in this area.

Sardinia samples (codes SRY and SRO) present an intermediate pattern of differentiation, with some samples from both “Slender” and “Stocky” morphs sharing the same sequence with the morphologically distinct phenotype. These identities on ITS-rDNA sequences were already observed within another genus within Parazoanthidae (*Antipathozoanthus*^43^). These Authors found that, despite being highly variable compared to mitochondrial markers (due to presence of indels), ITS marker can leave some uncertainties regarding the specific status of different morphotypes. ITS markers are therefore unable to fully resolve the phylogenetic relationships of this group^43^, which coincides with what was already described for other groups of cnidarians (for instance *Eunicella*^9,44^). Hypervariable ITS sequences can lead to alignment challenges (difference in both length and nucleotide identity), and intragenomic variation, that are widely acknowledged as being the primary obstacles to successfully using these sequences in phylogenetic inference in Zoanthidea^45^. These difficulties stress the need to increase the number of analyzed individuals to detect even small differences that can unlock the relationships among species, particularly in Zoanthidae phylogenetic studies^46^.

Despite these molecular issues, from the phylogenetic point of view, our results are in accordance with the latest accepted phylogeny of Parazoanthidae^24,25^ suggesting paraphyly of the genus *Parazoanthus.* As observed previously by^46^, the “Slender” *P. axinellae* is genetically similar to *P. anguicomus* and, to a lesser extent, to the Atlantic-Caribbean counterparts such as *Parazoanthus capensis*^47^*.* These are Atlantic species which are able to colonize sponges just like *P. axinellae*, but do not depend on such associations to survive^46^. The “Stocky” morphotype showed a genetic similarity with the shallow Pacific water *P. elongatus*, *P. juanfernandezii*^24,46–48^ and with the deep Atlantic *P. aliceae sp. n.*^24^. All these species are mainly found on rocks rather than on sponges, similarly to the “Stocky” morphotype, and conversely to the “Slender” morphotype. However, despite consistency between our molecular results with those by other Authors^24,43^, genetic data should be taken with caution since discrepancies between genetic and morphological data were already documented. From a morphological point of view, *P. elongatus* and *P. aliceae* sp. n. are quite different from the *P. axinellae* species complex, and do not seem to share similar morphological features, which contradicts the low COI and ITS gene divergence we observed. Specifically, Ocaña and Brito^16^ recently evidenced that *Parazoanthus elongatus* from Chile, should be placed into a different genus based on morphological data (e.g. the absence of special spirulae, the scarce presence of mineral particles in the ectoderm, and the large spirulae found in tentacles). This confirms once more that uncovering the origin of the “Stocky” morph in the western Mediterranean is a challenge. Future research using an integrative taxonomy approach^13^ could complement the results of the present study regarding the status of the Mediterranean *Parazoanthus* genus. In fact taxonomy using only molecular tools should not be a replacement of classical taxonomy, but findings derived from one approach should be used as a driver for further investigation for the other. A multidisciplinary approach should include (1) reproductive biology of both morphotypes in sympatry, (2) field experiments to evaluate substrate specificity^26^, (3) studies of chemodiversity^49^, (4) naturalistic studies^16^ and (5) more variable molecular markers (e.g. RAD sequencing^50^; Terzin et al. unpublished) and/or sequencing of the complete mitochondrial genome^51^.

The results of this work stress the importance of studies on species delimitation and connectivity for the implementation of effective plans for the conservation of coralligenous species. In fact, other studies have observed that in closely related species, with similar biological features, differences in the phylogeographic patterns can occur^10,52^. Finally, our results highlight occurence of hidden diversity within the flag-genus *Parazoanthus*, even in well-studied geographical areas, calling for a carefull taxonomic reevaluation of other key-species of the fragile endemic Mediterranean coralligenous ecosystem^53^.

## Methods

The original observation of the occurrence of two clearly distinguishable morphotypes of *Parazoanthus* was done by SCUBA diving in Portofino where, during the same dive, the “Slender” morphotype was observed in the deeper and in the shallower layers of a cliff, while the “Stocky” morphotype was found at an intermediate depth. Based on these preliminary results we developed a sampling design at a Mediterranean scale identifying sites where the occurrence on *Parazoanthus* was recorded (Fig. 1c). We identified 11 locations, and in six of them both morphotypes occurred in sympatry (Table 1). In each location up to 30 polyps per morphotype of *Parazoanthus* were collected by SCUBA diving, keeping a minimum distance of 2 m between polyps to avoid sampling of clones.

All samples were immediately fixed in 80% ethanol and refrigerated at 4ºC.

DNA was extracted from single polyps using EuroClone EuroGold tissue DNA mini kit. Mitochondrial COI fragment was amplified using species-specific designed primers COIpaxFwd (sequence 5′–3′: CGGTATGATAGGAACAGC), and COIpaxRev (sequence5′-3′:CGGGGTCAAAGAAGGTAGTG). A fragment of the nuclear DNA including 18S, ITS-1, 5,8Sa, ITS-2, and 28S (hereafter ITS) was amplified using zoanthid-specific primers described in^54^. PCR was performed in a final volume of 25 µl per sample and included: 5 µl GoTaq Flexi Buffer 1x (Promega), 4 µl MgCl 25 mM, 0.5 µl dNTPs 10 mM, 0.5 µl of each primer (10 mM), and one unit of GO Taq G2 Flexi DNA polymerase (Promega) and filled with nuclease free water to reach the volume. Amplifications were conducted on a GeneAmp 2,700 thermal cycler (Applied Biosystems) under the following conditions: a hold at 94° C for 3′ followed by 30 cycles of denaturation at 94 °C for 45″, annealing at a primer specific temperature (59 °C for COI and 50° C for ITS) for 1′ and extension at 72 °C for 2′, finishing with a final extension at 72° for 7′.

PCR products were checked in 1.5% agarose gel stained with Gelred (BIOTIUM) 1% after a 20′ 120 V electrophoresis. They were then sent to Macrogen Europe Inc. for purification and sequencing.

Field and experimental protocols were approved by the University of Bologna, Italy and were performed in accordance with its relevant guidelines and regulations. No permit was required for the collection of the species.

## Data analysis

### Sequence quality check and alignment

Each sequence was checked for quality in MEGA v.6 and good quality sequences were aligned using MAFFT (Multiple Alignment using Fast Fourier Transform)^55^ through *phyloch* R package^56^ (see below for more detail).

### Genetic diversity and structure among *Parazoanthus axinellae* samples

The number of haplotypes (H) and haplotype and nucleotide diversity (Hd and π respectively), were calculated for each sample and marker in DNAsp^57^. Haplotype networks for COI and ITS markers were built using *pegas* R package^35^ in Rstudio (Version 1.1.453)^58^. Haplotypes were reconstructed with haploNet function using an infinite site model based on Hamming distances of DNA sequences. As there was an overlap between genetically approximate haplotypes on the Fig. 2 (in particular for the ITS gene), alluvial diagrams were created using an online platform RAWGraphs^59^ to better clarify Morphotype/Haplotype/Sampling locations correlations, and Inkscape (version 0.92.4) was used to integrate the plots and finalize graph compilation.

A Minimum Spanning Tree was computed as in^60^ to infer links between the most similar haplotypes based on a previously computed distance matrix, and to visualize the number of mutations between them. This was done by performing a Multidimensional Scaling (MDS) analysis on Hamming distances computed between haplotypes, using the show.mutation = T option to show the number of mismatches between linked haplotypes. The results were presented as a two-dimensional MDS plot.

Genetic differentiation between samples (with morphs from the same locality treated as different populations) was estimated using Φ statistics (Φ_ST_ based on haplotype frequencies and molecular divergence) and its significance determined using a permutation test (10,000 permutations) for each marker in Arlequin v. 3.5^61^. Significance values were corrected for multiple comparisons following FDR correction method^62^.

Hierarchical Analysis of Molecular Variance (AMOVA) was carried out for each marker using (1) morphotype as grouping factor (two levels: “Slender” and ”Stocky”) and (2) biogeographic areas as grouping factor^38^: Gulf of Lion and Ligurian Sea (Banyuls-sur-Mer, Alassio, Portofino and Porto Venere), Tyrrhenian Sea (Giannutri, Olbia and Napoli), Ionian and South Adriatic (Gallipoli and Tremiti), and North Adriatic (Chioggia and Rovinj).

To study the demographic history of the two morphotypes *Fu and Li*^33^ and *Tajima*^63^′s neutrality tests were performed in DNAsp for each locus and morphotype across the study area. Subsequently, the demographic history was also assessed by performing a mismatch distribution analysis, in which the frequencies of pairwise nucleotide differences between individuals were compared with the expected values under a sudden expansion model^34^ using Arlequin. The best fit was tested by evaluating both the sum of squared deviation (SSD) and the Harpending’s raggedness (HRI) indexes with a total of 1,000 permutations.

### Phylogeny and differentiation within Family Parazoanthidae

We retrieved COI and ITS sequences belonging to the family Parazoanthidae from Genbank. Full details on the species, accession numbers, approximate geographic origin, and original reference are given in Supplementary Table S7 for each marker.

We decided to perform all downstream analyses at the taxonomic level of family due to large uncertainties regarding the phylogeny and systematics within Parazoanthidae at lower taxonomic categories, which led to significant and recent taxonomic modifications for these cnidarians (e.g.^25,64^). For each marker, the retrieved sequences were aligned together with our sequences using multiple sequence aligner MAFFT v7.310 (Multiple Alignment using Fast Fourier Transform)^55^ through *phyloch* R package^56^. *P. axinellae* + Genbank COI sequences were aligned with default parameters, whereas L-INS-i algorithm with 1,000 iterations (iterative refinement method incorporating local pairwise alignment information) was used to align *P. axinellae* + Genbank ITS sequences due to a presence of gaps between individuals showing high sequence divergence.

Gblocks Version 0.91b^65^ was then ran from R to exclude gaps between highly divergent sequences, with parameters finally set to: b1 = 0.8 (the minimum number of sequences for a conserved position), b2 = 0.9, (the minimum number of sequences for a flank position), b4 = 2, (the minimum length of a block, default 2) and b5 = "h", to remove gaps present in > 50% of individuals.

Likelihood ratio test was performed with modelTest function in *phangorn*^66,67^ to decide on the model of nucleotide evolution that best fits COI and ITS markers, and the best nucleotide substitution model was identified based on minimum AICc values for each marker separately.

Then, Bayesian inference (BI) in MrBayes v. 3.2.6^68^ and Maximum Likelihood (ML) trees with UPGMA algorithm and clustering dendrograms were used to resolve phylogenetic situation of *P. axinellae* species complex within Parazoanthidae family based on COI and ITS gene markers. Distance matrices were calculated using the suggested F81 nucleotide substitution model and Maximum Likelihood (ML) trees with 1,000 bootstrap iterations were estimated from the obtained distance matrices using the UPGMA algorithm. This was done in *phangorn*^66,67^ by computing the likelihood of a given tree with the function pml(), and with the function optim.pml(), which was used to optimize tree topology and branch length for F81 model of nucleotide evolution. Tree plotting was done using plotBS function, with haplotypes presented on the tree nodes in roman numerals, and bootstrap support values (based on 1,000 iterations) shown on tree branches with a cutoff value of *p* = 50 (%). To further clarify “haplotype-sampling location” associations, clustering dendrograms were reconstructed for each gene marker in RAWGraphs^59^ and combined with ML trees in Inkscape (version 0.92.4). Hierarchically, the clustering dendrograms are composed out of 2 levels for sequences downloaded from GenBank (Species code, Number of sequences) and 3 levels for individuals sampled in our study (Morphotype Color, Sampling Location, Number of sequences). Sequences originating from our samples were colored in yellow and orange for “Slender” and “Stocky” morphotypes, respectively, while all sequences downloaded from GenBank are in grey.

## Supplementary information

Supplementary Information.

Supplementary data 1.

Supplementary data 2.

## Data Availability

The dataset and the R codes supporting the conclusions of this article are available in fasta format as additional files in the Supplementary information.
