# Supplementary material for: Evidence of genetic isolation between two Mediterranean morphotypes of Parazoanthus axinellae
Source: Sci Rep. 2020 Aug 18;10:13938. doi: 10.1038/s41598-020-70770-z (PMC7434761; doi:10.1038/s41598-020-70770-z)
Supplement: Supplementary file 1 — Supplementary Information. [file 41598_2020_70770_MOESM1_ESM.pdf]

Evidence of genetic isolation between two Mediterranean morphotypes of *Parazoanthus axinellae*

Adriana Villamor[1,2], Lorenzo F. Signorini[1,3] , Federica Costantini[1,4*], Marko Terzin[1] & Marco Abbiati[1,5,6]

[1]Dipartimento di Scienze Biologiche, Geologiche ed Ambientali (BiGeA) & Centro Interdipartimentale di Ricerca per le Scienze Ambientali (CIRSA), Università di Bologna, Via S. Alberto 163, 48123 Ravenna, Italy.

[2] International Council for the Exploration of the Sea, HC Andersen 22-24, 1553 Copenhagen, Denmark.

[3] Faculty of Life Sciences, Tel Aviv University, Ramat Aviv 69978, Israel

[4] CoNISMa, Piazzale Flaminio 9, 00196 Rome, Italy.

[5] Consiglio Nazionale Delle Ricerche, Istituto di Scienze Marine, ISMAR, Bologna, Italy.

[6] Dipartimento di Beni Culturali, Università di Bologna, Via degli Ariani, 1, 48121 Ravenna, RA, Italy.

*Corresponding Author: federica.costantini@unibo.it

Figure S1. Minimum Spanning Tree was used to visualize links between reconstructed COI (above) and ITS (below) haplotypes, and to show the number of mutations between "adjacent" haplotypes for *Parazoanthus axinellae* species complex. Multidimensional scaling (MDS) analysis was performed in pegas R package to arrange haplotype sequences depending on their similarity, and based on the computed Hamming distances. The results were presented in a two-dimensional MDS plot. Haplotypes from the Figure S1 for each marker are identical to those in haplotype networks (Figure 2). Haplotypes are shown in roman numerals, with yellow and orange colours corresponding to "Slender" and "Stocky" morphotypes, respectively. The number of mutations between haplotypes is shown in a grey rectangle, and links inferred from the network are shown as blue lines, connecting the most similar haplotypes. Due to an overlap between highly similar sequences (in particular for the ITS marker), tables were presented on the right of the figure for COI and ITS genes to show the number of mutations (Mut) detected for each haplotype pair linked in the Minimum Spanning Tree (H1 and H2).

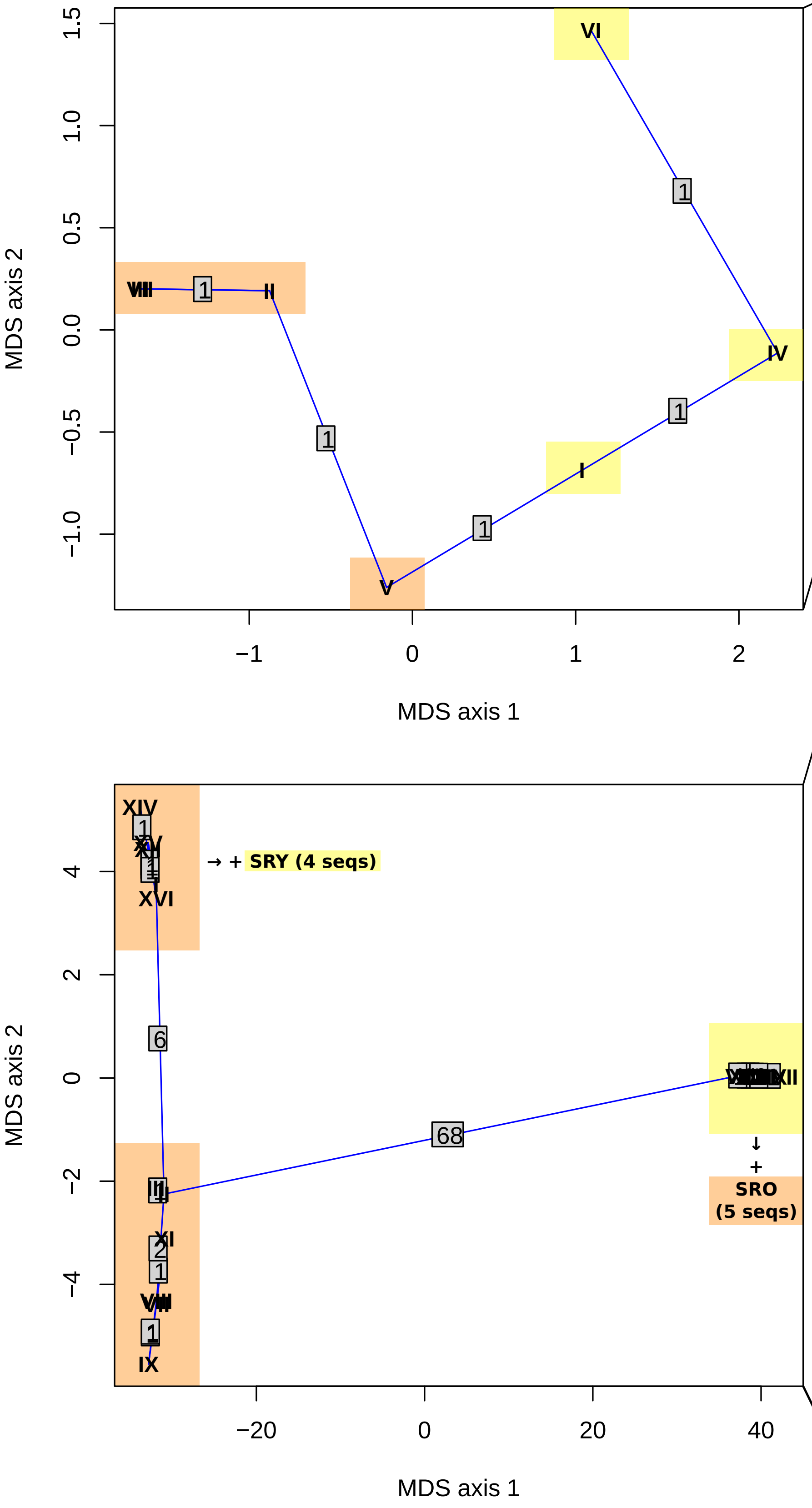

H1	H2	Mut
I	IV	1
I	V	1
II	III	1
II	V	1
II	VII	1
IV	VI	1

H1	H2	Mut
I	XII	1
I	XV	1
II	III	1
IV	V	1
IV	X	1
IV	XIII	1
IV	XVIII	1
IV	XX	1
V	VI	1
VII	IX	1
VII	XI	1
VIII	IX	1
XII	XIV	1
XV	XVI	1
XVII	XIX	1
II	VII	2
IV	XIX	2
I	II	6
II	VI	68

Figure S2. COI dataset: pairwise Φ_{ST} values between samples. Samples are labelled as in Table 1.

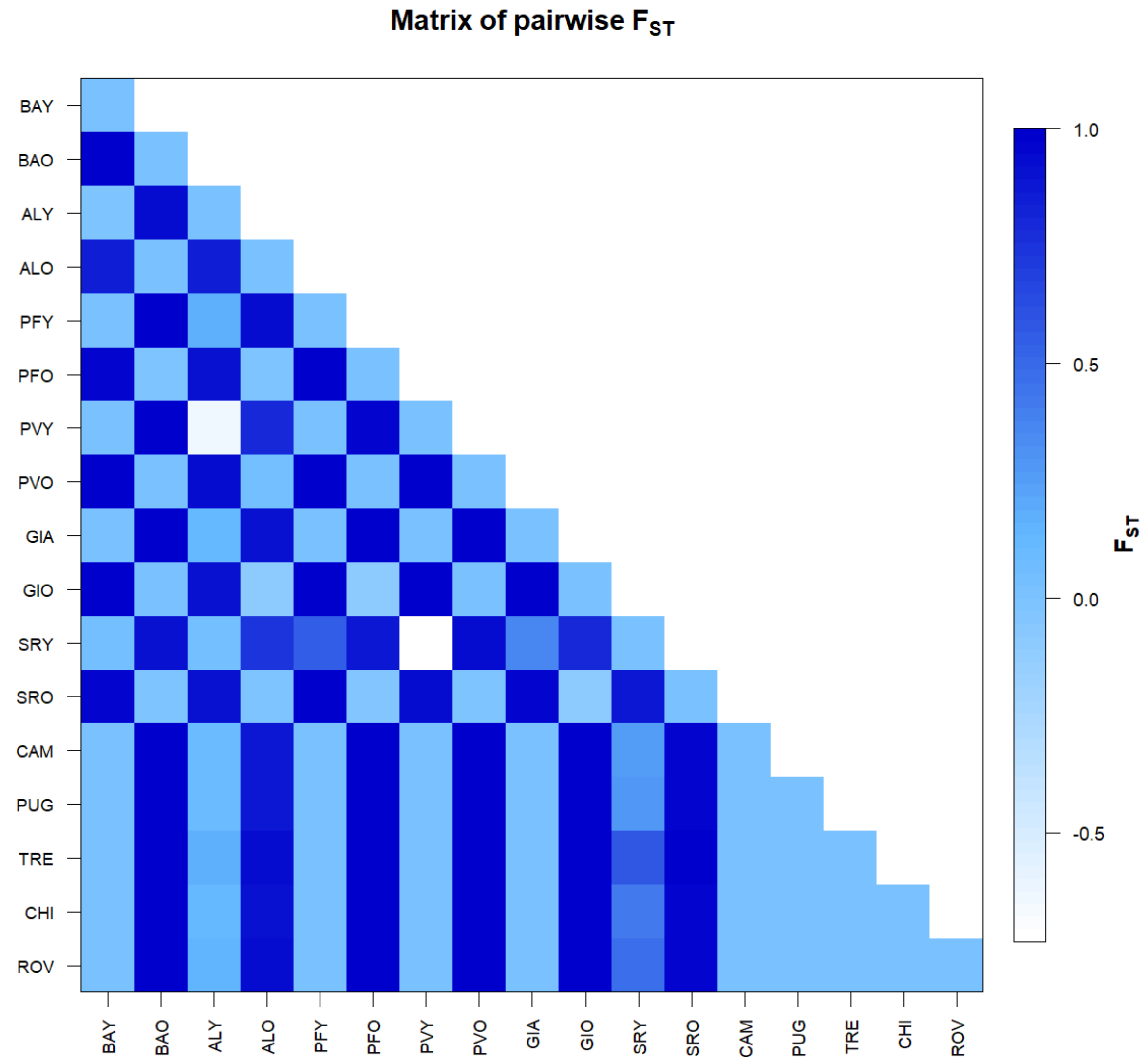

Figure S3. ITS dataset: pairwise Φ_{ST} values between samples. Samples are labelled as in Table 1.

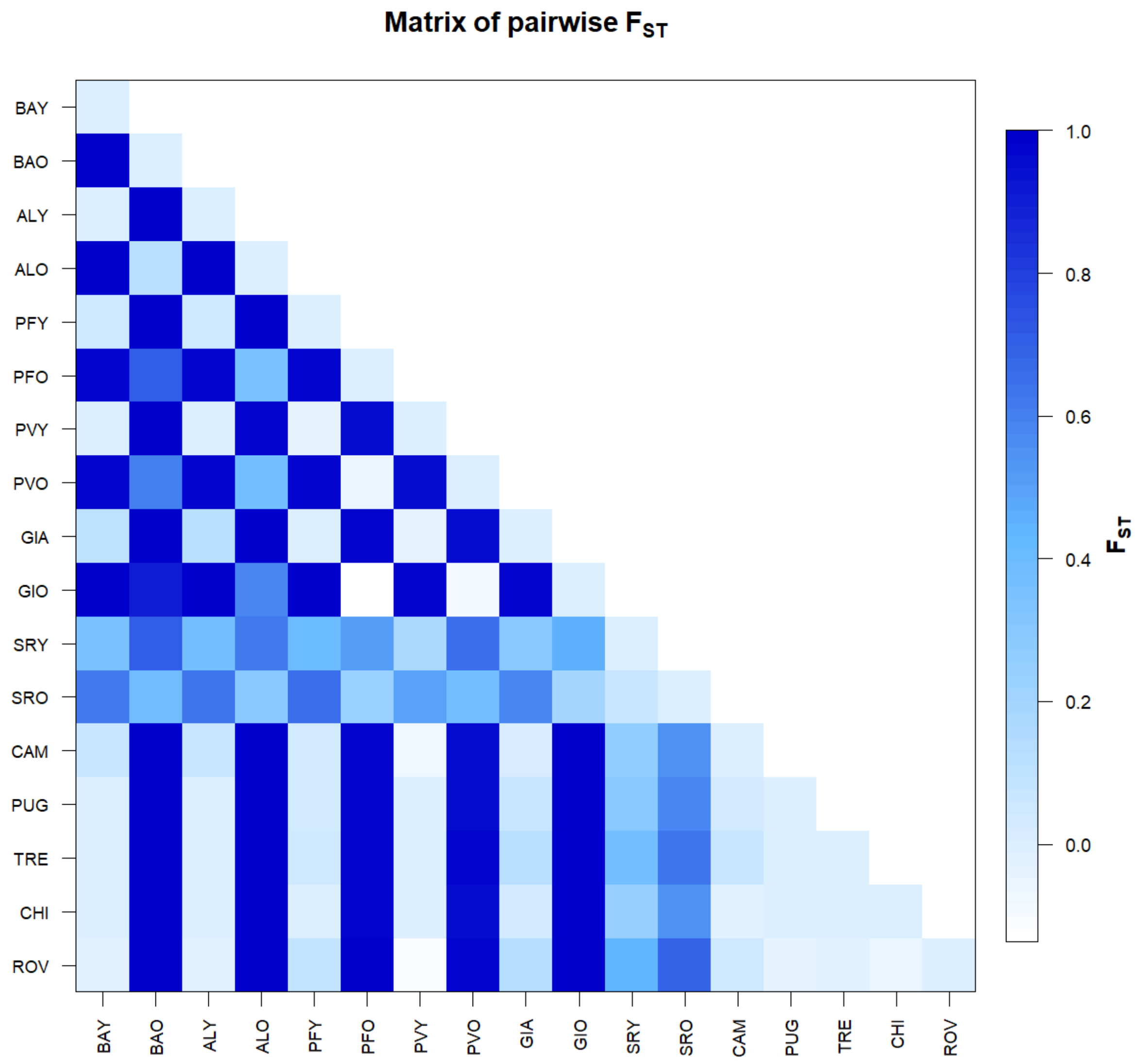

Figure S4. Mismatch distribution of pairwise differences in COI sequences (black bold line) compared to that expected under a sudden expansion scenario (black fine line). Two genetic pools can be identified, as shown by the secondary peak of larger pairwise differences.

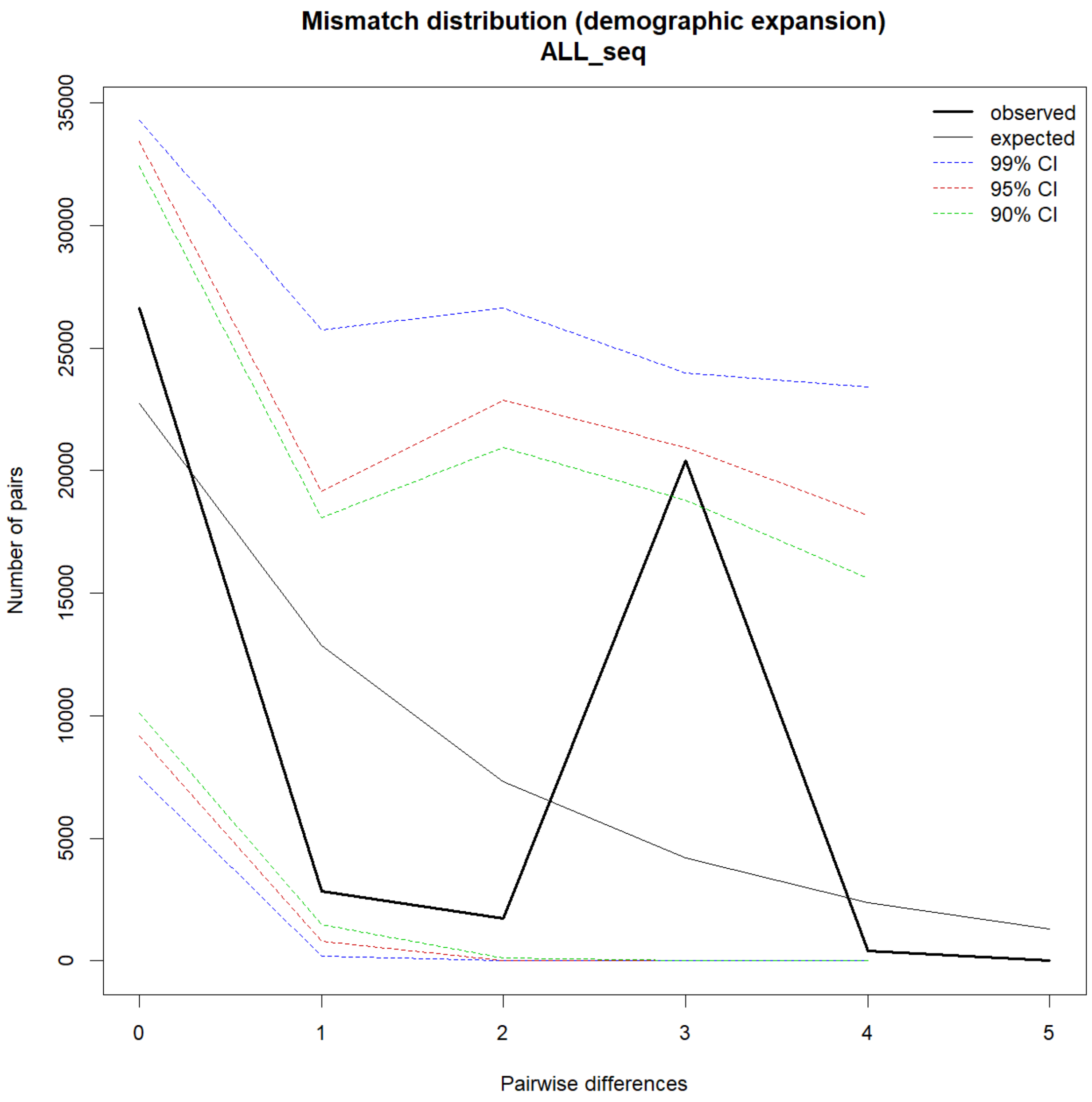

Figure S5. Mismatch distribution of pairwise differences in ITS sequences (red line) compared to that expected under a sudden expansion scenario (green line). At least two distant genetic pools can be identified, as shown by the secondary peak of larger pairwise differences, some intermediate peaks can also be seen closer to the vertical axis.

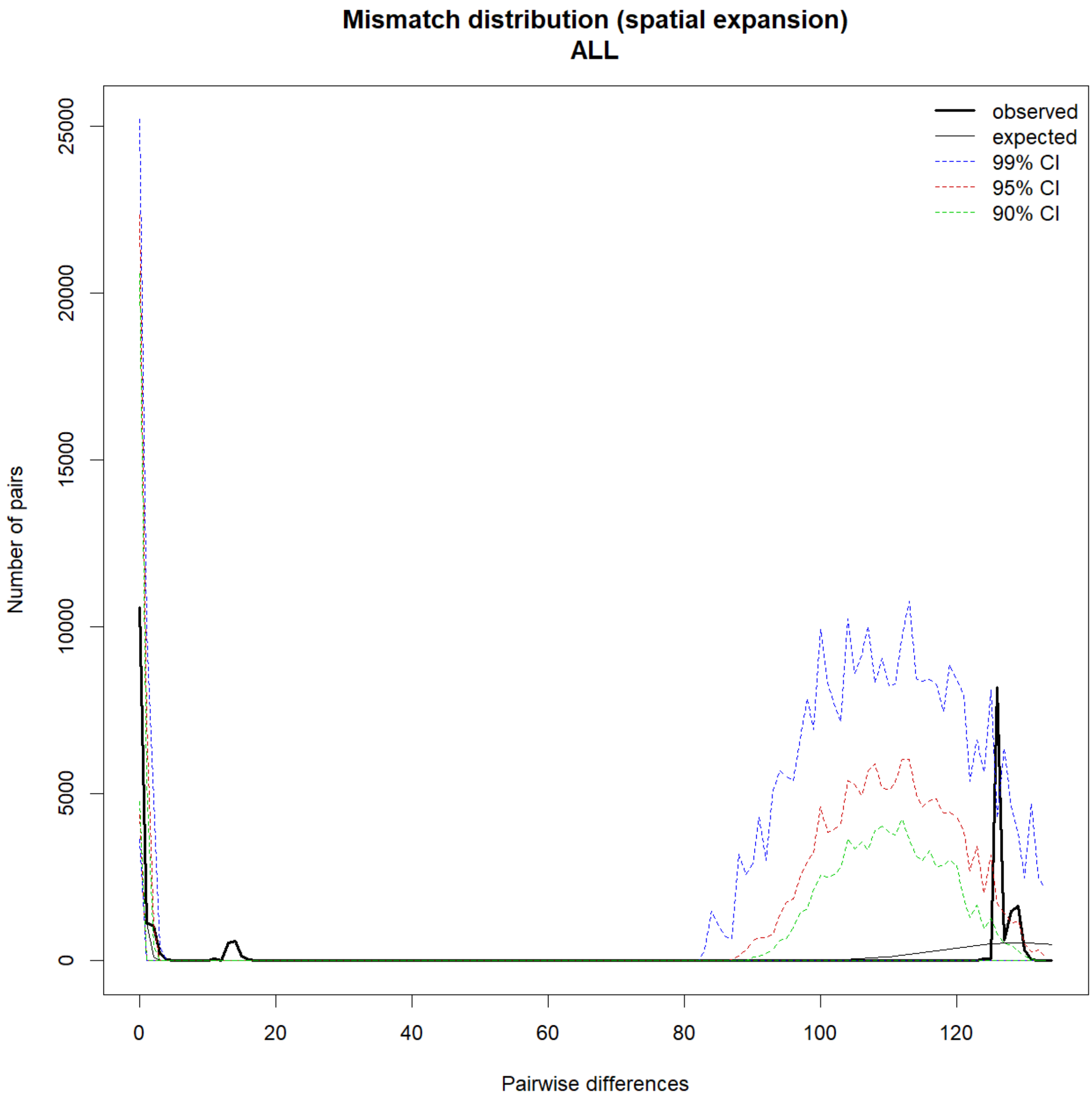

Table S1. Location-haplotype assignments in each of the 7 haplotypes for COI marker within *Parazoanthus axinellae* species complex.

	ALO	ALY	BAO	BAY	CAM	CHI	GIA	GIO	PFO	PFY	PUG	PVO	PVY	ROV	SRO	SRY	TRE
**I**	0	8	0	0	0	0	0	0	0	0	0	0	0	0	0	2	0
**II**	15	0	18	0	0	0	0	5	18	0	0	20	0	0	21	0	0
**III**	1	0	0	0	0	0	0	0	0	0	0	0	0	0	0	0	0
**IV**	0	33	0	4	11	21	17	0	0	39	12	0	1	26	0	3	42
**V**	1	0	0	0	0	0	0	0	0	0	0	0	0	0	1	0	0
**VI**	0	0	0	0	0	0	0	0	0	0	0	0	0	0	0	1	0
**VII**	0	0	0	0	0	0	0	0	0	0	0	0	0	0	1	0	0

Table S2. Location-haplotype assignments in each of the 20 haplotypes for ITS marker within *Parazoanthus axinellae* species complex.

	ALO	ALY	BAO	BAY	CAM	CHI	GIA	GIO	PFO	PFY	PUG	PVO	PVY	ROV	SRO	SRY	TRE
**I**	13	0	24	0	0	0	0	1	3	0	0	7	0	0	5	4	0
**II**	1	0	0	0	0	0	0	0	0	0	0	0	0	0	0	0	0
**III**	1	0	0	0	0	0	0	0	0	0	0	0	0	0	0	0	0
**IV**	0	16	0	15	8	8	10	0	0	17	11	0	5	24	5	3	16
**V**	0	0	0	0	0	1	0	0	0	0	0	0	0	0	0	0	0
**VI**	0	0	0	0	0	0	1	0	0	0	0	0	0	0	0	0	0
**VII**	0	0	0	0	0	0	0	3	2	0	0	5	0	0	0	0	0
**VIII**	0	0	0	0	0	0	0	0	1	0	0	0	0	0	0	0	0
**IX**	0	0	0	0	0	0	0	1	0	0	0	0	0	0	0	0	0
**X**	0	0	0	0	0	0	0	0	0	3	0	0	0	0	0	0	0
**XI**	0	0	0	0	0	0	0	0	0	0	0	8	0	0	0	0	0
**XII**	0	0	0	0	0	0	0	0	0	0	0	1	0	0	0	0	0
**XIII**	0	0	0	0	0	0	0	0	0	0	0	0	0	1	0	0	0
**XIV**	0	0	0	0	0	0	0	0	0	0	0	0	0	0	2	0	0
**XIX**	0	0	0	0	0	0	0	0	0	0	0	0	0	0	0	1	0
**XV**	0	0	0	0	0	0	0	0	0	0	0	0	0	0	1	0	0
**XVI**	0	0	0	0	0	0	0	0	0	0	0	0	0	0	1		0
**XVII**	0	0	0	0	0	0	0	0	0	0	0	0	0	0	0	1	0
**XVIII**	0	0	0	0	0	0	0	0	0	0	0	0	0	0	0	1	0
**XX**	0	0	0	0	0	0	0	0	0	0	0	0	0	0	0	1	0

Table S3. COI dataset: pairwise Φ_{ST} values between samples. Significance was assessed by permutation test. Significant values after correction for multiple testing are reported in bold. Samples are labelled as in Table 1.

	BAY	BAO	ALY	ALO	PFY	PFO	PVY	PVO	GIA	GIO	SRY	SRO	CAM	PUG	TRE	CHI	ROV
BAY	*																
BAO	**1**	*															
ALY	-0.01	**0.92**	*														
ALO	**0.84**	0.02	**0.85**	*													
PFY	0	**1**	**0.17**	**0.94**	*												
PFO	**0.97**	0	**0.91**	-0.01	**0.99**	*											
PVY	0	**1**	-0.65	**0.81**	0	**0.96**	*										
PVO	**1**	0	**0.92**	0.03	**1**	0	**1**	*									
GIA	0	**1**	0.11	**0.90**	0	**0.98**	0	**1**	*								
GIO	**1**	0	**0.89**	-0.08	**1**	-0.10	**1**	0	**1**	*							
SRY	0.04	**0.91**	0.03	**0.74**	0.56	**0.88**	-0.73	**0.92**	**0.36**	**0.81**	*						
SRO	**0.95**	0	**0.90**	-0.03	**0.98**	-0.04	**0.94**	0	**0.97**	-0.10	**0.87**	*					
CAM	0	**1**	0.08	**0.88**	0	**0.98**	0	**1**	0	**1**	**0.26**	**0.97**	*				
PUG	0	**1**	0.09	**0.89**	0	**0.98**	0	**1**	0	**1**	**0.28**	**0.96**	0	*			
TRE	0	**1**	**0.18**	**0.94**	0	**0.97**	0	**1**	0	**1**	**0.58**	**0.94**	0	0	*		
CHI	0	**1**	0.12	**0.91**	0	**0.98**	0	**1**	0	**1**	**0.41**	**0.97**	0	0	0	*	
ROV	0	**1**	**0.14**	**0.92**	0	**0.98**	0	**1**	0	**1**	**0.46**	**0.97**	0	0	0	0	*

Table S4. ITS dataset: pairwise Φ_{ST} values between samples. Significance was assessed by permutation test. Significant values after correction for multiple testing are reported in bold. Samples are labelled as in Table 1.

	BAY	BAO	ALY	ALO	PFY	PFO	PVY	PVO	GIA	GIO	SRY	SRO	CAM	PUG	TRE	CHI	ROV
BAY	*																
BAO	**1**	*															
ALY	0	**1**	*														
ALO	**0.99**	0.11	**0.98**	*													
PFY	0.08	**0.99**	0.08	**0.99**	*												
PFO	**0.98**	0.71	0.98	0.35	**0.98**	*											
PVY	0	**1**	0	**0.98**	-0.02	**0.96**	*										
PVO	**0.97**	**0.60**	0.97	**0.37**	**0.97**	-0.06	**0.95**	*									
GIA	0.10	**0.99**	0.11	**0.98**	-0.002	**0.97**	-0.09	**0.96**	*								
GIO	**0.99**	**0.90**	0.99	**0.58**	**0.99**	-0.14	**0.98**	-0.08	**0.98**	*							
SRY	0.35	**0.71**	0.36	**0.62**	0.40	0.51	0.18	0.65	0.29	0.46	*						
SRO	0.62	**0.39**	0.63	0.30	0.66	0.23	0.48	0.37	0.58	0.19	0.07	*					
CAM	0.06	**0.99**	0.07	**0.98**	0.02	**0.97**	-0.08	**0.96**	0.011	**0.98**	0.26	0.55	*				
PUG	0	**1**	0	**0.98**	0.03	**0.97**	0	**0.96**	0.07	**0.99**	0.30	0.58	0.02	*			
TRE	0	**1**	0	**0.98**	0.06	**0.98**	0	**0.97**	0.11	**0.99**	0.36	0.63	0.07	0	*		
CHI	0	**1**	0	**0.98**	0.03	**0.97**	0	**0.96**	0.03	**0.98**	0.25	0.54	-0.01	0	0	*	
ROV	-0.02	**0.99**	-0.02	**0.99**	0.09	**0.99**	-0.11	**0.97**	0.15	**0.99**	0.45	0.69	0.04	-0.04	-0.02	-0.06	*

Table S5. Species-haplotype assignments in each of the 16 haplotypes for COI marker within Parazoanthidae family.

	ALI	ALO	ALY	ANG	BAO	BAY	CAM	CHI	ELO	GIA	GIO	GRA	MES	PAR	PAX	PFO	PFY	PUG	PVO	PVY	ROV	SAV	SRO	SRY	SWI	TRE
**I**	0	0	8	1	0	0	0	0	0	0	0	0	0	1	0	0	0	0	0	0	0	0	0	2	0	0
**II**	0	0	33	0	0	4	11	21	0	17	0	0	0	0	1	0	39	12	0	1	26	0	0	3	0	42
**III**	0	0	0	0	0	0	0	0	0	0	0	0	0	0	0	0	0	0	0	0	0	0	0	1	0	0
**IV**	0	0	0	0	0	0	0	0	0	0	0	0	0	0	0	0	0	0	0	0	0	0	0	0	5	0
**V**	1	15	0	0	18	0	0	0	0	0	5	0	0	0	0	18	0	0	20	0	0	0	21	0	0	0
**VI**	0	1	0	0	0	0	0	0	0	0	0	0	0	0	0	1	0	0	0	0	0	0	1	0	0	0
**VII**	0	0	0	0	0	0	0	0	4	0	0	0	0	0	0	0	0	0	0	0	0	0	0	0	0	0
**VIII**	0	1	0	0	0	0	0	0	0	0	0	0	0	0	0	0	0	0	0	0	0	0	0	0	0	0
**IX**	0	0	0	0	0	0	0	0	0	0	0	0	0	0	0	0	0	0	0	0	0	0	1	0	0	0
**X**	0	0	0	0	0	0	0	0	0	0	0	0	0	0	0	0	0	0	0	0	0	1	0	0	0	0
**XI**	0	0	0	0	0	0	0	0	0	0	0	0	0	0	0	0	0	0	0	0	0	1	0	0	0	0
**XII**	0	0	0	0	0	0	0	0	0	0	0	0	3	0	0	0	0	0	0	0	0	0	0	0	0	0
**XIII**	0	0	0	0	0	0	0	0	0	0	0	1	0	0	0	0	0	0	0	0	0	0	0	0	0	0
**XIV**	0	0	0	0	0	0	0	0	0	0	0	2	0	0	0	0	0	0	0	0	0	0	0	0	0	0
**XV**	0	0	0	0	0	0	0	0	0	0	0	0	0	0	0	0	0	0	0	0	0	0	0	0	0	0
**XVI**	1	0	0	0	0	0	0	0	0	0	0	0	0	0	0	0	0	0	0	0	0	0	0	0	0	0

Table S6. Species-haplotype assignments in each of the 38 haplotypes for ITS marker within Parazoanthidae family.

	ALO	ALY	ANG	BAO	BAY	BEC	BEP	CAM	CAP	CHI	DAR	ELO	GIA	GIO	GRA	JUA	PAR	PAX	PFO	PFY	PUG	PVO	PVY	ROV	SRO
**I**	13	0	0	24	0	0	0	0	0	0	0	0	0	1	0	0	0	0	3	0	0	7	0	0	5
**II**	1	0	0	0	0	0	0	0	0	0	0	0	0	0	0	0	0	0	0	0	0	0	0	0	0
**III**	1	0	0	0	0	0	0	0	0	0	0	0	0	0	0	0	0	0	0	0	0	0	0	0	0
**IV**	0	16	0	0	15	0	0	8	0	8	0	0	10	0	0	0	0	9	0	17	11	0	5	24	5
**V**	0	0	1	0	0	0	0	0	0	0	0	0	0	0	0	0	0	0	0	0	0	0	0	0	0
**VI**	0	0	1	0	0	0	0	0	0	0	0	0	0	0	0	0	0	0	0	0	0	0	0	0	0
**VII**	0	0	0	0	0	2	0	0	0	0	0	0	0	0	0	0	0	0	0	0	0	0	0	0	0
**VIII**	0	0	0	0	0	0	1	0	0	0	0	0	0	0	0	0	0	0	0	0	0	0	0	0	0
**IX**	0	0	0	0	0	0	0	1	0	0	0	0	0	0	0	0	0	0	0	0	0	0	0	0	0
**X**	0	0	0	0	0	0	0	0	1	0	0	0	0	0	0	0	0	0	0	0	0	0	0	0	0
**XI**	0	0	0	0	0	0	0	0	0	0	2	0	0	0	0	0	0	0	0	0	0	0	0	0	0
**XII**	0	0	0	0	0	0	0	0	0	0	0	2	0	0	0	1	0	0	0	0	0	0	0	0	0
**XIII**	0	0	0	0	0	0	0	0	0	0	0	0	1	0	0	0	0	1	0	0	0	0	0	0	0
**XIV**	0	0	0	0	0	0	0	0	0	0	0	0	0	3	0	0	0	0	2	0	0	5	0	0	0
**XV**	0	0	0	0	0	0	0	0	0	0	0	0	0	0	1	0	0	0	0	0	0	0	0	0	0
**XVI**	0	0	0	0	0	0	0	0	0	0	0	0	0	0	1	0	0	0	0	0	0	0	0	0	0
**XVII**	0	0	0	0	0	0	0	0	0	0	0	0	0	0	0	0	1	0	0	0	0	0	0	0	0
**XVIII**	0	0	0	0	0	0	0	0	0	0	0	0	0	0	0	0	1	0	0	0	0	0	0	0	0
**XIX**	0	0	0	0	0	0	0	0	0	0	0	0	0	0	0	0	1	0	0	0	0	0	0	0	0
**XX**	0	0	0	0	0	0	0	0	0	0	0	0	0	0	0	0	1	0	0	0	0	0	0	0	0
**XXI**	0	0	0	0	0	0	0	0	0	0	0	0	0	0	0	0	0	0	1	0	0	0	0	0	0
**XXII**	0	0	0	0	0	0	0	0	0	0	0	0	0	0	0	0	0	0	1	0	0	0	0	0	0
**XXIII**	0	0	0	0	0	0	0	0	0	0	0	0	0	0	0	0	0	0	0	3	0	0	0	0	0
**XXIV**	0	0	0	0	0	0	0	0	0	0	0	0	0	0	0	0	0	0	0	0	0	8	0	0	0
**XXV**	0	0	0	0	0	0	0	0	0	0	0	0	0	0	0	0	0	0	0	0	0	1	0	0	0
**XXVI**	0	0	0	0	0	0	0	0	0	0	0	0	0	0	0	0	0	0	0	0	0	0	0	1	0
**XXVII**	0	0	0	0	0	0	0	0	0	0	0	0	0	0	0	0	0	0	0	0	0	0	0	0	2
**XXVIII**	0	0	0	0	0	0	0	0	0	0	0	0	0	0	0	0	0	0	0	0	0	0	0	0	1
**XXIX**	0	0	0	0	0	0	0	0	0	0	0	0	0	0	0	0	0	0	0	0	0	0	0	0	1
**XXX**	0	0	0	0	0	0	0	0	0	0	0	0	0	0	0	0	0	0	0	0	0	0	0	0	0
**XXXI**	0	0	0	0	0	0	0	0	0	0	0	0	0	0	0	0	0	0	0	0	0	0	0	0	0
**XXXII**	0	0	0	0	0	0	0	0	0	0	0	0	0	0	0	0	0	0	0	0	0	0	0	0	0
**XXXIII**	0	0	0	0	0	0	0	0	0	0	0	0	0	0	0	0	0	0	0	0	0	0	0	0	0
**XXXIV**	0	0	0	0	0	0	0	0	0	0	0	0	0	0	0	0	0	0	0	0	0	0	0	0	0
**XXXV**	0	0	0	0	0	0	0	0	0	0	0	0	0	0	0	0	0	0	0	0	0	0	0	0	0
**XXXVI**	0	0	0	0	0	0	0	0	0	0	0	0	0	0	0	0	0	0	0	0	0	0	0	0	0
**XXXVII**	0	0	0	0	0	0	0	0	0	0	0	0	0	0	0	0	0	0	0	0	0	0	0	0	0

**XXXVIII**	0	0	0	0	0	0	0	0	0	0	0	0	0	0	0	0	0	0	0	0	0	0	0	0	0

Table S7. Species, molecular marker, accession number, approximate geographic origin, and original reference of the retrieved sequences from Genbank belonging to the family Parazoanthidae. Also accession numbers of the sequences obtained in this study are indicated.

**Species**	**Marker**	**Accession number**	**Samplig location**	**Code**	**References**
Parazoanthus axinellae	COI	EF672659.2	NE Atlantic	PAX	Cachet, et al. (2015)
Parazoanthus axinellae	COI	MT769330 - MT769651	Mediterranean Sea	See Table 1 in the main manuscript	This study
Parazoanthus tunicans	COI	EF672667.1		TUN	Sinniger, Reimer and Pawlowski, unpublished
Parazoanthus parasiticus	COI	EF672663.1|		PAR	Sinniger, Reimer and Pawlowski, unpublished
Parazoanthus elongatus	COI	EF672662.1	South Pacific	ELO1	Sinniger, Reimer and Pawlowski, unpublished
Parazoanthus elongatus	COI	EF672661.1	South Pacific	ELO2	Sinniger, Reimer and Pawlowski, unpublished
Parazoanthus elongatus	COI	EU591622.1	Chile	ELO3	Sinniger & Häussermann (2009)
Parazoanthus elongatus	COI	EU591621.1	Chile	ELO4	Sinniger & Häussermann (2009)
Parazoanthus anguicomus	COI	EF672660.1	NE Atlantic	ANG	Sinniger, Reimer and Pawlowski, unpublished
Parazoanthus gracilis	COI	EF672668.1		GRA1	Sinniger, Reimer and Pawlowski, unpublished
Parazoanthus gracilis	COI	EU591629.1	South Pacific	GRA2	Sinniger & Häussermann (2009)
Parazoanthus gracilis	COI	EU591628.1	South Pacific	GRA3	Sinniger & Häussermann (2009)
Parazoanthus swiftii	COI	KT454364.1		SWI1	Santos et al. (2016)
Parazoanthus swiftii	COI	KT454363.1		SWI2	Santos et al. (2016)
Parazoanthus swiftii	COI	KT454362.1		SWI3	Santos et al. (2016)
Parazoanthus swiftii	COI	KT454361.1		SWI4	Santos et al. (2016)
Parazoanthus swiftii	COI	KR092589.1		SWI5	Montenegro et al. (2015)
Parazoanthus swiftii	COI	KJ794180.1		SWI6	Reimer et al. (2017)
Parazoanthus swiftii	COI	KJ794176.1		SWI7	Reimer et al. (2017)
Parazoanthus aliceae	COI	KJ459372	Portugal	ALI1	Carreiro Silva et al. (2017)
Savalia savaglia	COI	AB247356.1	Spain: Canary Islands, Gran Canaria	SAV1	Reimer et al. (2017)

Savalia savaglia	COI	HQ110947.1	Spain	SAV2	Altuna et al. (2010)
Mesozoanthus fossii	COI	EF672655.1	South Pacific	MES1	Sinniger, Reimer and Pawlowski, unpublished
Mesozoanthus fossii	COI	EF672654.1	South Pacific	MES2	Sinniger, Reimer and Pawlowski, unpublished
Mesozoanthus fossii	COI	EF672653.1	South Pacific	MES3	Sinniger, Reimer and Pawlowski, unpublished
Epizoanthus paguricola	COI	AB247347.1	Outgroup	EPI	Reimer et al. (2007)
Bergia catenularis (ex *Parazoanthus catenularis*)	ITS	189343743	Caribbean Sea	BEC2	Swain (2009)
Bergia catenularis (ex *Parazoanthus catenularis*)	ITS	189343744	Caribbean Sea	BEC2	Swain (2009)
Bergia puertoricense (ex *Parazoanthus puertoricense*)	ITS	189343768	Caribbean Sea	BEP	Swain (2009)
Umimayanthus parasiticus (ex *Parazoanthus parasiticus*)	ITS	189343750	Caribbean Sea	PAR1	Swain (2009)
Umimayanthus parasiticus (ex *Parazoanthus parasiticus*)	ITS	189343751	Caribbean Sea	PAR2	Swain (2009)
Umimayanthus parasiticus (ex *Parazoanthus parasiticus*)	ITS	189343752	Caribbean Sea	PAR3	Swain (2009)
Umimayanthus parasiticus (ex *Parazoanthus parasiticus*)	ITS	189343754	Caribbean Sea	PAR4	Swain (2009)
Parazoanthus swiftii	ITS	189343787	Caribbean Sea	SWI1	Swain (2009)
Parazoanthus swiftii	ITS	189343788	Caribbean Sea	SWI2	Swain (2009)
Parazoanthus swiftii	ITS	189343789	Caribbean Sea	SWI3	Swain (2009)
Parazoanthus swiftii	ITS	189343790	Caribbean Sea	SWI4	Swain (2009)
Parazoanthus swiftii	ITS	189343791	Caribbean Sea	SWI5	Swain (2009)
Parazoanthus swiftii	ITS	189343792	Caribbean Sea	SWI6	Swain (2009)
Parazoanthus swiftii	ITS	189343793	Caribbean Sea	SWI7	Swain (2009)
Parazoanthus swiftii	ITS	299801616	Caribbean Sea	SWI8	Sinniger et al. (2010)
Parazoanthus swiftii	ITS	299801617	Caribbean Sea	SWI9	Sinniger et al. (2010)
Parazoanthus swiftii	ITS	299801618	Caribbean Sea	SWI10	Sinniger et al. (2010)
Parazoanthus swiftii	ITS	672354155	South Atlantic	SWI11	Reimer et al.. (2017)

Parazoanthus swiftii	ITS	672354156	South Atlantic	SWI12	Reimer et al.. (2017)
Parazoanthus swiftii	ITS	808352396	Gulf of Mexico	SWI13	Montenegro et al. (2015)
Hydrozoanthus tunicans (ex *Parazoanthus tunicans*)	ITS	189343794	Caribbean Sea	TUN1	Swain (2009)
Hydrozoanthus tunicans (ex *Parazoanthus tunicans*)	ITS	195547262	Caribbean Sea	TUN2	Sinniger & Häussermann (2009)
Hydrozoanthus tunicans (ex *Parazoanthus tunicans*)	ITS	195547264	Caribbean Sea	TUN3	Sinniger & Häussermann (2009)
Hydrozoanthus gracilis (ex *Parazoanthus gracilis*)	ITS	195547265	Indian Ocean	GRA1	Sinniger & Häussermann (2009)
Hydrozoanthus gracilis (ex *Parazoanthus gracilis*)	ITS	300498436	North Pacific	GRA2	Swain (2010)
Parazoanthus capensis	ITS	300498430	Port Elizabeth, South Africa	CAP	Swain (2010)
Parazoanthus darwini	ITS	171027784	Galapagos	DAR1	Reimer et al. (2008)
Parazoanthus darwini	ITS	171027785	Galapagos	DAR2	Reimer et al. (2008)
Parazoanthus elongatus	ITS	195547239	New Zealand	ELO1	Sinniger & Häussermann (2009)
Parazoanthus elongatus	ITS	195547240	South Pacific	ELO2	Sinniger & Häussermann (2009)
Parazoanthus juanfernandezii	ITS	300498428	South Pacific	JUA	Swain (2010)
Parazoanthus anguicomus	ITS	195547249	NE Atlantic	ANG1	Sinniger & Häussermann (2009)
Parazoanthus anguicomus	ITS	300498429	NE Atlantic	ANG2	Swain (2010)
Parazoanthus axinellae	ITS	195547245	NW Mediterranean Sea	PAX1	Sinniger & Häussermann (2009)
Parazoanthus axinellae	ITS	189343733	Adriatic Sea	PAX2	Swain (2009)
Parazoanthus axinellae	ITS	189343734	Adriatic Sea	PAX3	Swain (2009)
Parazoanthus axinellae	ITS	189343735	Adriatic Sea	PAX4	Swain (2009)
Parazoanthus axinellae	ITS	189343736	Gulf of Mexico	PAX5	Swain (2009)
Parazoanthus axinellae	ITS	189343737	NW Mediterranean Sea	PAX6	Swain (2009)
Parazoanthus axinellae	ITS	189343738	NW Mediterranean Sea	PAX7	Swain (2009)
Parazoanthus axinellae	ITS	675382184	NW Mediterranean Sea	PAX8	Reimer et al.(2008)
Parazoanthus axinellae	ITS	675382188	NE Atlantic	PAX9	Sinniger & Häussermann (2009)
Parazoanthus axinellae	ITS	758818030	NW Mediterranean Sea	PAX10	Cachet, et al. (2015)

Parazoanthus axinellae	ITS	MT771056-MT771287	Mediterranean Sea	See Table 1 in the main manuscript	This study
Epizoanthus spp	ITS		Outgroup		

References:

Altuna, Á., Sinniger, F., & Aldrey, J. M. Occurrence of *Savalia savaglia* (Anthozoa: Zoantharia) in the Ría de Arousa (Galicia, north-western Spain, north-eastern Atlantic). *Mar. Biod. Rec.* **3** (2010).

Cachet, N., et al. Metabolomic profiling reveals deep chemical divergence between two morphotypes of the zoanthid *Parazoanthus axinellae*. *Sci. Rep.* **5**, 8282 (2015).

Montenegro, J., Sinniger, F., & Reimer, J. DUnexpected diversity and new species in the sponge-Parazoanthidae association in southern Japan. *Mol. Phyl. Evol*. **89**, 73-90 (2015).

Reimer, J. D., Lorion, J., Irei, Y., Hoeksema, B. W., & Wirtz, P. Ascension Island shallow-water Zoantharia (Hexacorallia: Cnidaria) and their zooxanthellae (*Symbiodinium*). *J. Mar. Biol. Ass. UK* **97**, 695-703 (2017).

Reimer, J. D., Nonaka, M., Sinniger, F., & Iwase, F. Morphological and molecular characterization of a new genus and new species of parazoanthid (Anthozoa: Hexacorallia: Zoantharia) associated with Japanese Red Coral. *Coral Reefs* **27** 935 (2008).

Reimer, J. D., Sinniger, F., & Hickman, C. P. Zoanthid diversity (Anthozoa: Hexacorallia) in the Galapagos Islands: a molecular examination. *Coral Reefs*, **27**, 641-654 (2008).

Santos, M. E. A., Kitahara, M. V., Lindner, A., & Reimer, J. D. Overview of the order Zoantharia (Cnidaria: Anthozoa) in Brazil. Mar. Biod. **46**, 547-559 (2016).

Sinniger, F., & Häussermann, V. Zoanthids (Cnidaria: Hexacorallia: Zoantharia) from shallow waters of the southern Chilean fjord region, with descriptions of a new genus and two new species. *Org. Div. Evol.* **9**, 23-36 (2009).

Sinniger, F., Reimer, J. D., & Pawlowski, J. The Parazoanthidae (Hexacorallia: Zoantharia) DNA taxonomy: description of two new genera. Mar. Biodiv. **40**, 57-70 (2010).

Swain, T. D. Phylogeny-based species delimitations and the evolution of host associations in symbiotic Zoanthusids (Anthozoa, Zoanthusidea) of the wider Caribbean region. *Zool. J. Lin. Soc.* **156**, 223-238 (2009).

Swain, T. D. Evolutionary transitions in symbioses: dramatic reductions in bathymetric and geographic ranges of Zoanthidea coincide with loss of symbioses with invertebrates. *Mol. Ecol.* **19**, 2587-2598 (2010).
